# Macroscopic extent of gastric mucosal atrophy: increased risk factor for esophageal squamous cell carcinoma in Japan

**DOI:** 10.1186/1471-230X-9-34

**Published:** 2009-05-18

**Authors:** Tomoyuki Akiyama, Masahiko Inamori, Hiroshi Iida, Hiroki Endo, Kunihiro Hosono, Kyoko Yoneda, Koji Fujita, Masato Yoneda, Hirokazu Takahashi, Ayumu Goto, Yasunobu Abe, Hiroyuki Kirikoshi, Noritoshi Kobayashi, Kensuke Kubota, Satoru Saito, Yasushi Rino, Atsushi Nakajima

**Affiliations:** 1 Gastroenterology Division, Yokohama City University School of Medicine, 3-9 Fukuura, Kanazawa-ku, Yokohama, Japan; 2 Surgery Division, Yokohama City University School of Medicine, 3-9 Fukuura, Kanazawa-ku, Yokohama, Japan

## Abstract

**Background:**

We aimed to estimate whether the macroscopic extent of gastric mucosal atrophy is associated with a risk for esophageal squamous cell carcinoma using a case-control study in Japanese subjects, a population known to have a high prevalence of CagA-positive *H. pylori *infection.

**Methods:**

Two hundred and fifty-three patients who were diagnosed as having esophageal squamous cell carcinoma, and 253 sex- and age-matched controls were enrolled in the present study. The macroscopic extent of gastric mucosal atrophy was evaluated based on the Kimura and Takemoto Classification. A conditional logistic regression model with adjustment for potential confounding factors was used to assess the associations.

**Results:**

Body gastritis, defined endoscopically, was independently associated with an increased risk for esophageal squamous cell carcinoma.

**Conclusion:**

Our findings suggest that macroscopic body gastritis may be a risk factor for esophageal squamous cell carcinoma in Japan. Further studies are needed to confirm these findings.

## Background

Esophageal cancer is the world's eighth most common malignancy, affecting approximately 500,000 individuals worldwide each year [1]. In Japan, the age-standardized mortality rate of this cancer in 2000 was 10.4/100,000 for men, approximately eight times that of women, and the sixth most frequent cause of death from cancer in Japanese men [2]. There are two major histological types of esophageal cancer, squamous cell carcinoma and adenocarcinoma, and their epidemiological features differ considerably. Esophageal squamous cell carcinoma (ESCC) has a predilection for black and Asian populations and, worldwide, more than 80% of esophageal cancers are squamous cell carcinomas. In contrast, esophageal adenocarcinoma affects white populations predominantly. The frequency of squamous cell carcinoma in Western countries has declined while there has been a dramatic rise in the frequency of esophageal adenocarcinoma over the past several decades [3,4]. Meanwhile more than 90% of esophageal cancers in Japan have been squamous cell carcinoma, and no significant changes have been identified [5].

Recently, a large population-based case-control study in Sweden has demonstrated that cytotoxin-associated gene A (CagA)-positive *H. pylori *infection is an increased risk factor for ESCC, and gastric mucosal atrophy (GMA) may be an important mediator of the positive association between CagA seropositivity and ESCC [6]. These findings suggest that GMA, induced CagA-positive *Helicobacter pylori *(*H. pylori*) infection, is a risk factor for ESCC in Sweden. Iijima K et al. revealed that GMA, defined histologically or serologically, was associated with the risk for ESCC and the risk seemed to increase with the progression of GMA in Japanese subjects [7].

In the present study, we investigated whether the macroscopic extent of GMA is associated with a risk for ESCC using a case-control study in Japanese subjects, a population known to have a high prevalence of CagA-positive *H. pylori *infection.

## Methods

### Patients

Two hundred and fifty-three consecutive patients who were diagnosed as having ESCC at the Gastroenterology Division of Yokohama City University Hospital from January 1997 to September 2008 were retrospectively enrolled in the present study. Exclusion criteria were the inability to obtain complete profiles from the subjects' medical records; destruction of the esophagogastric junction (EGJ) by advanced ESCC; the inability to observe the stomach on upper endoscopy because of esophageal stenosis due to advanced ESCC, or the subject had previously undergone an upper digestive tract operation.

For each case, a control matched by sex and age group was randomly selected from among patients who had undergone endoscopies as part of a health checkup during the same period and who had no endoscopically-observed localized lesions in the upper gastrointestinal tract. Exclusion criteria for the controls were the inability to obtain their complete profiles from their medical records; or they had previously undergone an upper digestive tract operation.

### Endoscopic findings

Our hospital operates a digital filing system for endoscopic images. All digital endoscopic images were independently and retrospectively reviewed by two trained endoscopists to investigate the endoscopic findings, including GMA, hiatal hernia, erosive esophagitis, and Barrett's epithelium. If there was any inconsistency in the assessment of the digital endoscopic images, a final diagnosis was decided upon by a joint review of the digital endoscopic images.

### Gastric mucosal atrophy

**Figure 1:**
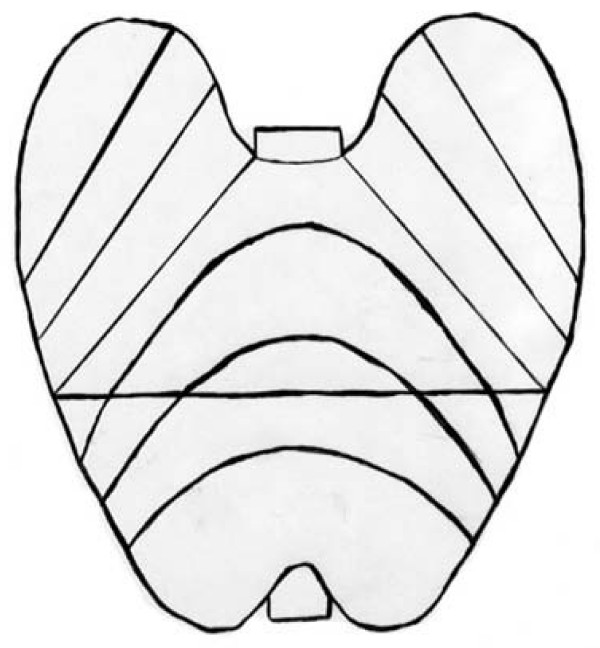
**Classification of an endoscopically evident atrophic pattern**. The atrophic border is the boundary between the pyloric and fundic gland territories, which is endoscopically recognized by discriminating differences in the color and height of the gastric mucosa. Cases of closed-type GMA have an atrophic boundary between the fundic mucosa and the pyloric mucosa in the antrum or lesser curvature of the gastric body. Cases of open-type GMA have an atrophic boundary in the lateral wall or greater curvature of the gastric body [8]. C, closed; O, open.

**Figure 2:**
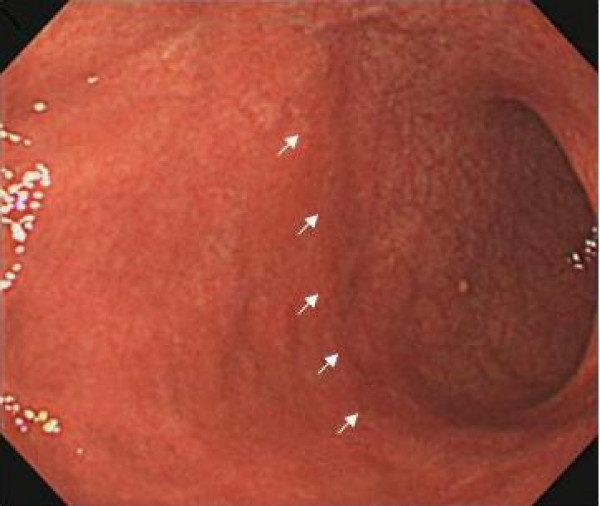
**An endoscopic image of open-type GMA**. The atrophic border, determined by discriminating differences in the color and height of the gastric mucosa, is recognized in the greater curvature of the angular portion. (White arrows indicate the atrophic border).

The atrophic area of the stomach that can be visualized endoscopically is known to extend from the antrum to the body. Previously, Kimura and Takemoto endoscopically divided GMA into six groups (C1, C2, C3, O1, O2, and O3; C, closed; O, open) (Figure 1, 2) [8]. GMA has been shown to progress from C1 to O3 successively, and this classification correlates well with the histological features of GMA [8]. Gastric acid secretion in patients with open-type GMA has been reported to be lower than that in patients with closed-type GMA [9]. In the present study, we defined O2–3 cases as body gastritis.

### Hiatal hernia

Hiatal hernia was diagnosed when the distance between the gastroesophageal junction and the diaphragmatic hiatus was 2 cm or more.

### Erosive esophagitis

Erosive esophagitis was diagnosed based on the Los Angeles Classification [10] and was divided into three groups: none, mild (grades A and B), or severe (grades C and D).

### Barrett's epithelium

The presence of Barrett's epithelium was diagnosed based on the C & M Criteria [11]. According to the criteria, Barrett's epithelium is defined as the macroscopic identification, using a standard endoscopy exam, of abnormal columnar esophageal epithelium suggestive of a columnar-lined distal esophagus. The length of Barrett's epithelium is measured (in centimeters) using the circumferential extent (the C extent) and the maximum extent (the M extent) above the gastroesophageal junction, identified as the proximal margin of the gastric mucosal folds [11].

### Patients profiles

Complete patient information at the time of the initial diagnosis, including age, sex, body mass index (BMI), regular drinking and smoking habits, was obtained from each patient's medical records.

### Ethics

The study was conducted in accordance with the Declaration of Helsinki. The study protocol was approved by the Ethics Committee of Yokohama City University Hospital.

### Statistical analysis

In a case control study, distribution of the demographic characteristics and related factors were compared between ESCC patients and controls. The statistical analysis included a Chi-square test or a Fisher exact test to compare percentages and a Mann-Whitney U test to compare continuous data. Various risk factors were also evaluated simultaneously using logistic regression. The level of significance was defined as p < 0.05. All statistical analyses were performed using Stat View software (SAS Institute, Cary, N.C.).

## Results

The comparison of patient profiles and endoscopic findings between the ESCC and the sex- and age-matched control groups is shown in Table 1. The majority of subjects were male, and median (range) age was 65 (38–86) years old both in the ESCC and control groups. The BMI in ESCC cases was significantly lower than in controls, suggests decreasing dietary intake caused by dysphagia and appetite loss in advanced ESCC subjects. The prevalences of current regular drinking and current smoking habits, and open-type 2–3 GMA were significantly higher in ESCC cases than in controls. There was no significant difference in the frequency of hiatal hernia between cases and controls. The rates of erosive esophagitis and Barrett's epithelium, as complications of gastroesophageal reflux disease (GERD), were significantly lower in ESCC cases than in controls.

**Table 1:** Comparison of Characteristics between ESCC cases and controls (P value: Mann-Whitney U test; *chi square test; **Fisher's exact test).

	ESCC cases (N = 253)	Controls (N = 253)	p-value
Patients profiles			
Male/Female	225/28	225/28	>0.9999
Age (median; range)	65; 38–86	65; 38–86	>0.9999
BMI (median; range)	20.9; 14.8–30.8	22.8; 14.5–33.1	<0.0001
Current regular drinker	210 (83.0%)	142 (56.1%)	<0.0001*
Current smoker Endoscopic findings	205 (81.0%)	133 (52.6%)	<0.0001*
GMA Open-type 2, 3	164 (64.8%)	127 (50.2%)	0.0009*
Hiatal hernia	60 (23.7%)	74 (29.2%)	0.1584*
Erosive esophagitis			
Total (LA-A to D)	13 (5.1%)	64 (25.3%)	<0.0001*
Mild (LA-A, B)	12 (4.7%)	59 (23.3%)	<0.0001*
Severe (LA-C, D)	1 (0.4%)	5 (2.0%)	0.2160**
			
Barrett's epithelium	80 (31.6)	114 (45.1%)	0.0019*

BMI = body mass index; GMA = gastric mucosal atrophy

Multiple logistic regression analysis of the clinical factors associated with ESCC is demonstrated in Table 2. After adjustment for clinical factors, including BMI, current regular drinking and smoking habits, open-type 2–3 GMA had an independently significant positive association with the prevalence of ESCC, which strongly suggests that macroscopic body gastritis was an independent risk factor for ESCC. Erosive esophagitis showed an independently significant inverse association with ESCC, while Barrett's epithelium had no such association.

**Table 2:** Multiple logistic regression analysis of clinical factors associated with ESCC.

Clinical factors	Odds ratio	95% confidence interval	P-value
Body mass index	0.870	0.813–0.930	<0.0001
Regular drinking habit	3.228	2.028–5.138	<0.0001
Smoking habit	3.231	2.062–5.063	<0.0001
GMA	1.572	1.035–2.386	0.0339
Hernia	0.928	0.575–1.498	0.7608
Erosive esophagitis	0.178	0.089–0.359	<0.0001
Barrett's epithelium	0.671	0.434–1.039	0.0734

## Discussion and conclusion

GMA induced by *H. pylori *infection may form a milieu that favors bacterial overgrowth which, in turn, may increase endogenous nitrosation [12,13], which was statistically significantly correlated with mortality from esophageal cancer, predominantly ESCC, in a Chinese ecologic study [14]. Thus, *H. pylori *infection may increase the risk of ESCC through abolishment of normal gastric secretion. However, there have been only a few studies regarding the relation between *H. pylori *infection and the risk of ESCC, and this issues remains controversial [6,15-17]. Some studies, using serum IgG antibodies against *H. pylori *as the only marker for the infection, demonstrated no or an inverse association between infection and ESCC [6,15-17]. On the other hand, one of these studies, using serum antibodies against the CagA of *H. pylori *or a combination of *H. pylori *IgG antibodies and CagA antibodies to define the infection, revealed a positive correlation between the two factors, and additionally found that GMA, assessed by serum levels of pepsinogen, was associated with an increased risk for ESCC [6]. There may have been an underestimation of the infection rate in the ESCC patients in these studies, in whom extensive GMA with spontaneous eradication of *H. pylori *might have occurred due to the hostile gastric condition to the bacterium [18,19].

The prevalence of *H. pylori *infection is much higher in Japan than Western countries [20]. Moreover, most strains of *H. pylori *isolated from Japanese patients are cagA-positive [21], which induces more severe inflammation and atrophy in the gastric mucosa, leading to changes in gastric acid secretion [22,23]. Several investigators have shown that *H. pylori *infection increases acid secretion in duodenal ulcers with antral-dominant gastritis [24,25], while it decreases acid secretion in body gastritis [26,27]. Furthermore, it has been confirmed that increased or decreased acid secretion is restored following the eradication of *H. pylori *[24-27], strongly supporting the premise that *H. pylori *infection alters the function of gastric acid secretion. In Japan, the incidence of GMA extending to the gastric body increases with age, resulting in a marked reduction of acid secretion in the elderly [27,28]. Iijima K et al. revealed that GMA, defined histologically or serologically, was associated with the risk for ESCC and the risk seemed to increase with the progression of GMA in Japanese subjects [7], suggesting that GMA may be an important mediator of the association between CagA-positive *H. pylori *and ESCC. This finding is consistent with a recent population-based case-control study from Sweden [6]. We did not unfortunately perform histological and serological diagnosis of GMA in the present study, in place of which the endoscopic diagnosis of body gastritis was adopted. In our study, endoscopic body gastritis, defined by open-type 2–3 GMA, was a independent risk factor for ESCC among Japanese subjects after adjustment for conventional risk factors, such as current regular drinking and smoking habits. This finding was consistent with the aforesaid subgroup analysis [6,7].

According to the univariate analysis, both erosive esophagitis and Barrett's epithelium, as complications of GERD, had an inverse-association with ESCC. Body gastritis, considered as a negatively associated factor of GERD, may be an important mediator of this association. But, even after adjustment for GMA Open-type 2–3, erosive esophagitis was independently significantly associated with ESCC. Advanced ESCC may play a partly suppressive role in the development of GERD through decreasing the dietary intake caused by dysphagia and appetite loss. It might be necessary to set up the ESCC cases, consisting only of superficial ESCC, for close assessment of extensive GMA as an important mediator of the correlation between ESCC and GERD complications. Our study has one potential limitation that may need to be considered, which is the difficulty in the selection of appropriate clinical controls, although this always occurs in case-control design studies. In the present study, we selected control subjects from among our outpatients who had undergone endoscopies for a health checkup during the same period and who had no endoscopically-identified localized lesions in the upper gastrointestinal tract. Nonetheless, the prevalences of endoscopic findings, including GMA, erosive esophagitis and Barrett's epithelium, in the controls were similar to those in previous reports with a Japanese population [29,30]. Hence, a bias in the selection of controls is unlikely, although a prospective cohort study is required to resolve this issue. In conclusion, body gastritis, diagnosed endoscopically, is an independent risk factor for ESCC in Japanese subjects. The identification of a high risk group for ESCC by macroscopic body gastritis, as well as heavy smoking and drinking, may be helpful in developing more efficient screening programs. Further studies are needed to confirm the causal relationship between GMA and the development of ESCC.

## Abbreviations

GMA: gastric mucosal atrophy; ESCC: esophageal squamous cell carcinoma; BMI: body mass index; LA: the Los Angeles Classification; C extent: circumferential extent; M extent: maximum extent; SSBE: short-segment Barrett's esophagus; LSBE: long-segment Barrett's esophagus;

## Competing interests

The authors declare that they have no competing interests.

## Authors' contributions

TA analyzed the upper endoscopies, collected the clinical data and wrote the manuscript, with contributions from MI. AN was responsible for the design of the study and collected the clinical data. MI performed the statistical analyses. TA and MI analyzed the upper endoscopies and participated in the design and coordination of the study. All authors read and approved the final manuscript.

## Pre-publication history

The pre-publication history for this paper can be accessed here:

http://www.biomedcentral.com/1471-230X/9/34/prepub
